# The correlation between IgM and IgG antibodies with blood profile in patients infected with severe acute respiratory syndrome coronavirus

**DOI:** 10.1186/s12948-022-00180-1

**Published:** 2022-12-22

**Authors:** Zahra Alibolandi, Amirreza Ostadian, Saeed Sayyah, Hamed Haddad Kashani, Hassan Ehteram, Hamid Reza Banafshe, Mohammad Hajijafari, Mahdi Sepehrnejad, Narjes Riahi Kashani, Mohammd-Javad Azadchehr, Hossein Nikzad, Elahe Seyed Hosseini

**Affiliations:** 1 Anatomical Sciences Research Center, Institute for Basic Sciences, Kashan University of Medical Sciences, Kashan, Iran; 2 Gametogenesis Research Center, Institute for Basic Sciences, Kashan University of Medical Sciences, Kashan, Iran; 3 Department of Pathology, School of Medicine, Kashan University of Medical Sciences, Kashan, Iran; 4 Department of Pharmacology, School of Medicine, Kashan University of Medical Sciences, Kashan, Iran; 5 Department of Anesthesiology, Beheshti Hospital, Kashan University of Medical Sciences, Kashan, Iran; 6 Department of Biostatistics, Infectious Disease Research Center, Kashan University of Medical Sciences, Kashan, Iran

**Keywords:** COVID-19, SARS-CoV-2, Illness severity, IgG, IgM

## Abstract

**Objectives:**

This study aimed to determine the levels of IgM and IgG antibody response to the severe acute respiratory syndrome coronavirus (SARS-CoV)-2 in coronavirus disease 2019 (COVID-19) patients with different disease severity.

**Methods:**

IgM and IgG antibody levels were evaluated via enzyme-linked immunosorbent assay (ELISA). In total, 100 patients with confirmed SARS-CoV-2 infection were enrolled in this study and viral RNA was detected by using Real-time PCR technique. Clinical and laboratory data were collected and analyzed after hospital admission for COVID-19 and two months post-admission.

**Results:**

The level of anti-SARS-CoV-2 antibody IgG was significantly higher in the severe patients than those in moderate and mild groups, 2 months after admission. Also, level of IgG was positively associated with increased WBC, NUT and LYM counts in sever than mild or moderate groups after admission to hospital.

**Conclusion:**

Our findings suggested that patients with severe illness might experience longer virus exposure times and have a stronger antibody response against viral infection. Thus, they have longer time immunity compared with other groups.

## Introduction

**Fig. 1:**
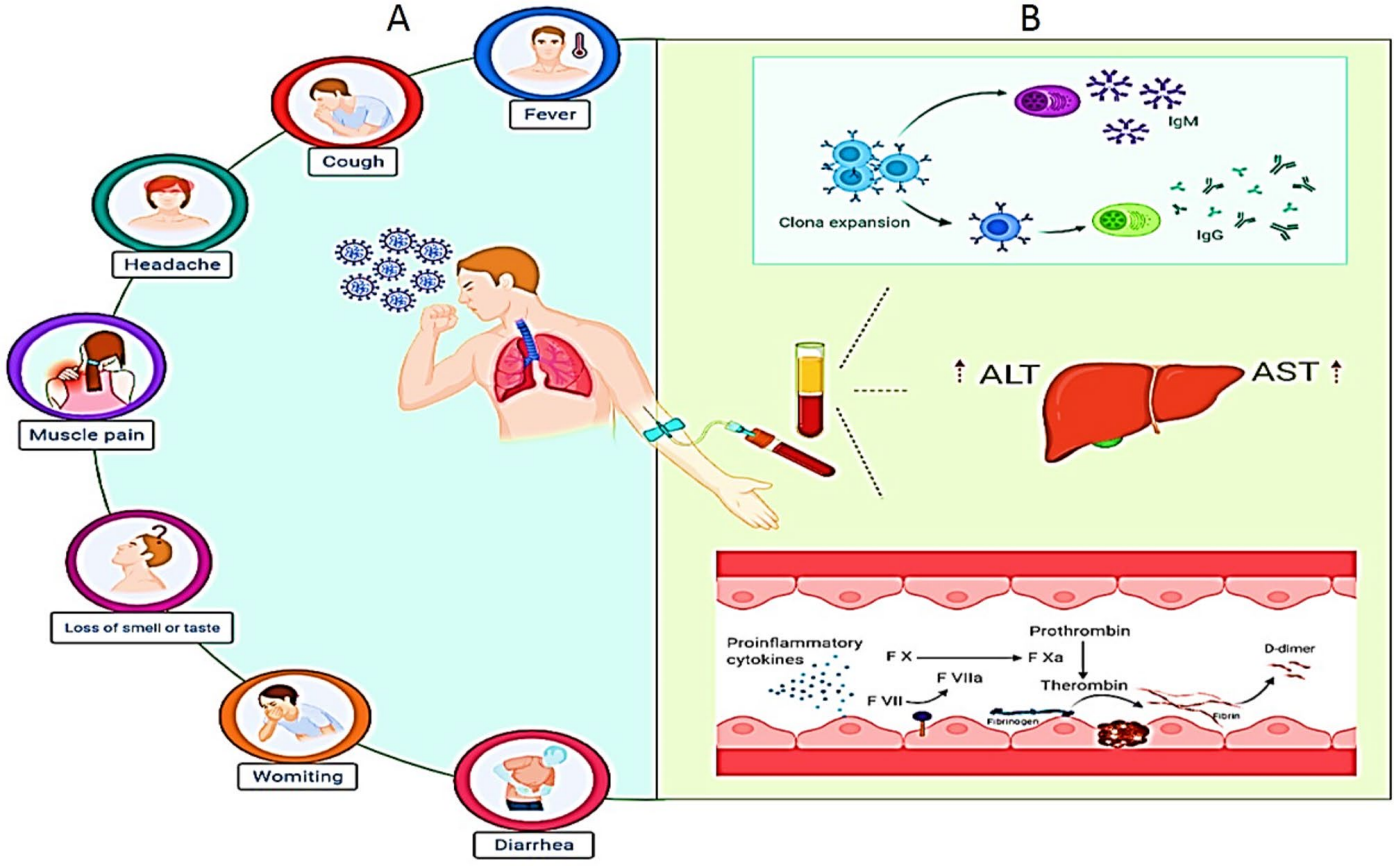
A. Some symptoms of COVID 19. B. Production of IgG and IgM in patient with COVID 19 and coagulation process (fibrinogen and D-dimer as markers in covid19)

The novel coronavirus, severe acute respiratory syndrome coronavirus (SARS-CoV)-2, has been identified as the causative pathogen of coronavirus disease 2019 (COVID-19) [1–3]. On 30 January 2020, World Health Organization (WHO) declared the outbreak of COVID-19 as a public health emergency of international concern [4]. Since December 2019, this serious disease has spread from China to more than 200 countries and territories worldwide via human-to-human transmission (Fig. 1) [5, 6]. The numbers of daily infected cases and COVID-related deaths are still increasing. As of 6 April 2020, a total of 103,528,865 confirmed cases and 2,237,799 deaths worldwide, also 1,417,999 cases and 57,959 deaths in Iran has been reported according to WHO. Clinical manifestations, CT imaging and a few laboratory tests have been commonly used for diagnosis of COVID-19 [6]. Currently, the laboratory diagnosis of SARS-CoV-2 is carried out by detecting viral RNA in throat or nasal swab specimens using real-time reverse transcription polymerase chain reaction (RT-PCR) assays [7]. A SARS-CoV-2 positive RT-PCR test is yielded after early onset of symptoms when the viral load and infectiousness gradually increases [7, 8]. Furthermore, this test is able to indicate only the presence of viral RNA in the specimens and not the amount of viable viruses or severity of the disease [9]. It is also notable that PCR positivity does not necessarily mean infectivity, since some cases shows positive result by real-time PCR even weeks after disease symptoms have been completely eradicated [10, 11]. In addition, after SARSCoV-2 infection, viral RNA may be undetectable after two weeks because its level is rapidly decreased [12]. However, a high percentage of false negative and false positive results is due to different factors (including the low accuracy of RT-qPCR kits, experimental conditions, and operation protocols, the sample quality and low viral load) affecting the sensitivity and accuracy of the RT-PCR test in the diagnosis of COVID-19 disease [13–19]. To diminish this problem, a combination of two techniques including PCR and Non-PCR based procedures is needed for accurate and rapid detecting of COVID-19. SARS-CoV-2 shares similar genetic and epidemiological features of previous SARS-CoV and MERS-CoV [1, 20]. Thus, a serologic test used for the detection of antibodies IgM/IgG generated against these COVs viruses may be useful to provide information about SARS-CoV-2 infection or even the time course of the infection in suspected patients (Fig. 1) [21, 22]. Consequently, a coupled detection of SARS-CoV-2 RNA and immunoglobulin G (IgG) may improve the performance of any of the two methods alone, also can increase the diagnostic accuracy [23, 24]. In this way, when a patient has negative results by RT-qPCR test for SARS-CoV-2, a positive serologic test and IgG detection is helpful to improve or confirm the diagnosis, also efficiently compensate the false negative limitations of RT-PCR tests [16, 25]. Therefore, the IgM and IgG antibodies produced by immune system become the main and most accurate procedure to detect a resolved or even past infection [12, 16]. Most recently, developed serological tests for virus specific IgM and IgG antibodies against SARS-CoV-2 have been recommended by the newest ‘Guideline of diagnosis and treatment for COVID-19’ issued by the Chinese National Health Commission [7, 26]. Most COVID-19 patients experience a mild illness and recovered quickly after appropriate clinical intervention. Whereas, some COVID-19 patients develop severe acute respiratory disease, multiple organ failure and even death over a short period of time [27–29]. Previous studies have reported that massive inflammatory responses induce the over activity of T cells, and leads to severe immune injury during SARS-CoV-2 infection [30]. However, the humoral immune response to COVID-19 is still greatly unknown. Here, we investigated IgG and IgM antibodies against SARS-CoV-2 detected by enzyme-linked immunosorbent assay (ELISA) in hospitalized patients during their course of disease. We also checked the humoral immunity responses of the patients two months after admission in the hospital based on disease severity.

## Methods

We carried out a cross sectional retrospective study of data and medical records of 100 COVID-19 patients admitted to Shahid beheshti Hospital of Kashan University of medical science from June 21 to September 20, 2020. COVID-19 infection confirmed based on symptoms and positive RT-PCR results of nasopharyngeal and throat swabs specimens. Patients included from 18 to 75 age old, Patients with cancer or immune disorder deficiency was excluded from this study. Serum levels of IgM-IgG antibodies targeting SARS–CoV–2 were tested upon patient admission and 2 months later. Patients were classified to three groups using the following criteria: (1) mild cases: Patients without manifestation of pneumonia on imaging; (2) moderate cases: Patients who had radiological findings of pneumonia, maybe show fever, respiratory symptoms and other symptoms; (3) and severe cases: Patients who had any one of these symptoms; Acute respiratory distress syndrome (ARDS), respiratory failure requiring mechanical ventilation, hypoxia (SpO_2_ ≤ 93%), shock or other organ failure that requires ICU care. The clinical information of all groups was collected from the medical records of the patients.

### Real-time RT-PCR

Throat swab or nasal swab specimens from the upper respiratory tract of all patients admitted to hospital for COVID-19 were collected and maintained in viral transport medium. Sputum specimens were also collected in some patients. SARS-CoV-2 infection was confirmed using TaqMan One-Step RT-PCR kits from Pishtaz Biotechnology Co., Ltd (Tehran, Iran), approved by the Iran Food and Drug Administration (FDA). In this method, the open reading frame 1ab (ORF1ab) and nucleocapsid protein (N) were simultaneously amplified and tested by RT-PCR.

### SARS-CoV-2 antibody detection

The IgM and IgG antibodies generated against SARS-CoV-2 in serum specimens were detected using IgG and IgM kits (Ideal Tashkhis Co. Tehran, Iran), according to the manufacturer’s instructions. The recombinant antigens contain nucleoprotein and spike protein of SARS-CoV-2. In this IgG and IgM kits 0.9 cut off index. The results ≥ 0.9 were reactive (positive), and the results < 0.9 were nonreactive (negative).

### Statistical analysis

The results are presented as mean ± standard deviation (SD) or median and interquartile range. Differences among groups were analyzed using the Mann–Whitney U-test. Statistical analyses were performed using Graph-Pad Prism version 6 (Graph-Pad Software Inc., San Diego, CA, USA). Statistical significance was determined to be *P* < 0.05.

## Results

### Clinical Symptom and characterize of patients with COVID-19

The present study included a total of 100 hospitalized patients (52(52%) male and 48 (48%) female) with confirmed COVID-19. The patients were classified into three clinical groups: mild (22 cases, 22%), moderate (38 cases, 38%) and sever (40 cases, 40%). The average age was 50 years (IQR, 37.25-58.75). The median ages in the severe (51.5 ± 13.5 years) and moderate (52.5 ± 12.5 years) groups were slightly higher than the mild group (43 ± 12.5 years). Thus, there was no significant association between patient ages with disease severity. Also, the percentage of males (57.5%) was somewhat more than females (42.5%) in the severe and moderate groups compared to the mild group (males 41%, females 59%) (Table 1). Moreover, radiological sign of pneumonia was observed at in 78 patients (78%) at the first clinical evaluation. The most common underlying diseases among the patients were diabetes 37(37%), hypertension 32(32%), cardiovascular disease 15(15%), asthma 14(14%). Some clinical symptoms of patients such as obesity (BMI > 30) (*p* = 0.05), fever (℃>39.0) (*p* = 0.009), shortness of breath (*p* = 0.015), muscle soreness (*p* = 0.007), chill (*p* = 0.025), loss of consciousness (*p* = 0.041), odor disorder (*p* = 0.036), anorexia (*p* = 0.029) and taste disorder (*p* = 0.027) were significantly associated with severity of disease. However, we find no significant correlation between age, sex, blood group and the underlying disease such as diabetic, hypertension, cardiovascular disease, organ failure, asthma disease, also other symptoms such as fatigue, sore throat, chest pain, headache, nausea, vomiting, diarrhea, visual impairment in patients with severity of disease (Table 1).

**Table 1:** Baseline characteristics of 100 COVID-19 patients

	No (%) Total (N = 100)	Mild (N = 22)	Moderate (N = 38)	Severe (N = 40)	*p*-value
Age, y, median (IQR)	50 (37.25-58.75)	43 (35.5–50)	52.5 (37.75–59.25)	51.5 (38.25–62.5)	0.198
Sex Female Male	48 (48%) 52 (52%)	13 (59%) 9 (41%)	18 (47.3%) 20 (52.7%)	17 (42.5%) 23 (57.5%)	0.455
Normal(BMI < 25) Overweight(25 < BMI < 30) obesity(BMI > 30)	22 (22%) 40 (40%) 38 (38%)	11 (50%) 5 (22.7%) 6 (27.3%)	5 (13.2%) 20 (52.6%) 13 (34.2%)	6 (15%) 15 (37.5%) 19 (47.5%)	0.05
Blood group A^+^ A^−^ B^+^ B^−^ AB^+^ AB^−^ O^+^ O^−^	29(29%) 4(4%) 24(24%) 2(2%) 11(11%) 1(1%) 28 (28%) 1(1%)	5 (23%) 0 4 (18%) 1(4.5%) 3 (14%) 1(4.5%) 7 (32%) 1(4.5%)	11 (29%) 2 (5%) 10 (26%) 1(2.6%) 5 (13%) 0 9 (23%) 0	13 (32.5%) 2(5%) 11(27.5%) 0 3 (7.5%) 0 12 (30%) 0	0.386
Diabetic	37 (37%)	5 (22.7%)	16 (42.1%)	16 (40%)	0.286
Hypertension	32 (32%)	4 (18%)	11 (29%)	17 (42.5%)	
Cardiovascular disease	15 (15%)	3 (13.6%)	3 (7.9%)	9 (22.5%)	0.192
Organ failure	25 (25%)	4 (18.2%)	11(28.9%)	10 (25%)	0.65
Asthma	14 (14%)	4 (18.2%)	4 (10.5%)	6(15%)	0.685
**Signs and symptoms**					
Fever (℃) < 37.3 37.3-39.0 > 39.0	37 (37%) 51 (51%) 12 (12%)	15 (68%) 6 (27%) 1 (4.5%)	13 (34%) 21 (55%) 4 (10%)	9 (22.5%) 24 (60%) 7 (17.5%)	0.009
SPO2 95-100% 90–94% < 90	37(37%) 52(52%) 11(11%)	22 (100%)	13(34%) 24(63%) 1(3%)	2(5%) 28(70%) 10 (25%)	
Fatigue	87 (87%)	20 (90.9%)	32 (84.2%)	35 (87.5%)	0.753
Muscle soreness	78 (78%)	17 (77.3%)	24 (63.2%)	37 (92.5%)	0.007
Chills	81 (81%)	18 (81.8%)	26 (68.4%)	37 (92.5%)	0.025
Sore throat	36 (36%)	8 (36%)	14 (37%)	14 (35%)	0.985
Shortness of breath	64 (64%)	10 (45%)	22 (58%)	32 (80%)	0.015
Cough	69 (69%)	16 (73%)	24 (63%)	29 (72.5%)	0.613
Chest pain	50 (50%)	8 (36.4%)	19 (50%)	23 (57.5%)	0.281
Headache	52 (52%)	14 (63.6%)	22 (57.9%)	16 (40%)	0.133
Nausea	36 (36%)	9 (40.9%)	14 (36.8%)	13 (32.5%)	0.797
vomiting	19 (19%)	5 (22.7%)	7 (18.4%)	7 (17.5%)	0.876
diarrhea	37 (37%)	9 (40.9%)	11(28.9%)	17 (42.5%)	0.423
Loss of consciousness	17 (17%)	0	7 (18.4%)	9 (22.5%)	0.041
Visual impairment	22 (22%)	2 (9.1%)	8 (21.1%)	12 (30%)	0.161
Odor disorder	53 (53%)	13(59.1%)	14 (36.8%)	26 (65%)	0.036
Anorexia	61 (61%)	9 (40.9%)	23 (60.5%)	30 (75%)	0.029
Abdominal pain	22 (22%)	7 (31.8%)	7 (18.4%)	8 (20%)	0.446
Taste disorder	39(39%)	6(27.3%)	11(28.9%)	22(55%)	0.027

Several laboratory markers such as ferritin, LDH and D-dimer were increased, while fibrinogen level was decreased in most patients at baseline after admission in hospital (step1) compare to two months after admission for COVID-19 (step2). Liver injury marker such as AST, ALT and ALP were also significantly increased at baseline (Table 2). In addition, we found that the higher WBC, NUT counts as well as lower LYM counts were correlated with the increased level of IgG two months after admission, resulting a longer time of immunity. It follows that the patients in sever group has better and longer immunity compare to mild groups (Table 3).

**Table 2:** Laboratory examinations of corona virus infected patients

		Mild			Moderate			Severe		
Blood routine	Normal range	step1	step2	*p*-value	step1	step2	*p*-value	step1	step2	*p*-value
WBC, ×10^9^/L	4_9	11.2 (6.07–13.99)	5.50 (4.65–7.35)	0	8.85 (5.44–11.38)	5.85 (5.27–6.60)	0.001	12.13 (9.39-16.00)	6.80 (5.57–7.97)	0
Neutrophils, ×10^9^/L	2.8_6.3	7.48(3.93–11.4)	3(2.42–4.53)	0	6.28(2.99–9.48)	3.34(2.7–3.8)	0	10.73(7.42–13.85)	3.75(2.9–5.12)	0
Lymphocytes, ×10^9^/L	2_4.5	1.32(0.9–2.11)	1.95(1.5–2.5)	0.05	1.33(0.89–1.91)	2.05(1.77–2.5)	0.001	0.93(0.77–1.39)	2.1(1.6–2.7)	0
Platelets, ×10^9^/L	150_450	238.5(188.5–262)	233(192.25-263.25)	0.974	198.5(149.25–269.5)	255.5(185-267.5)	0.056	223.5(180.25–294)	239(216.25-275.25)	0.023
AST (IU/L)	< 40	35(28.75–47.25)	28.5(24-32.25)	0.008	36(26-47.5)	28.5(25.75-33.00)	0.001	44.5(30.25–74.5)	30(28.00-32.75	0
ALT (IU/L)	< 40	30(22.25–40.25)	20.5(18.00-24.5)	0.006	28(20.5–66.00)	26(22.25-30.00)	0.012	54.5(25.25–74.5)	28(26–30)	0
ALP (IU/L)	65_306	178(153.25–241)	202.5(200.00-210.25)	0.426	176(131.25–203.00)	195(166.00-216.25)	0.032	194.5(147.25–245.00)	200(177.75–211.50)	0.914
LDH	< 500	394(313.5–508.0)	255(241.75–278.5)	0	470(385.00-534.75)	288.5(257.75–316.00)	0	466.5(378.75-657.75)	310(277.75–360.00)	0
D-dimer	> 200	50(50–287)	50(50–50)	0.007	200(50–300)	50(50–50)	0	325(50–800)	50(50–50)	0
Fibrinogen	200_400	484(403–484)	484(476.75-490.75)	0.001	403(356–484)	484(463.75–489.00)	0	403(323–440)	465.5(460–484)	0
Ferittin	10_200	271.44(117.99–573.50)	66.5(43.0-164.5)	0	363.05(172.66-937.49)	83(36.75-150.75)	0	546.66(304.75-942.24)	94(68.25-164.75)	0
IgG	< 0.9	0(0.00-2.92)	12.17(6.5–16.4)	0.116	0(0.00-0.37)	15.07(8.09–17.88)	0.05	0(0.00-6.67)	16.8(12.10-18.72)	0.001
IgM	< 0.9	0(0.00-1.35)	0(0–0)	0.5	0(0–0)	0(0.00-1.87)	0.1	0(0.00-1.35)	0(0–0)	0.1

Continuous variables are reported as median (interquartile range)

**Table 3:** Pearson correlation analysis between IgM-IgG antibody and laboratory profiles

	*p-*value	
	**IgM1**	**IgG2**
**WBC, ×10** ^**9**^ **/L**	0.69	0.027*
**Neutrophils, ×10** ^**9**^ **/L**	0.568	0.012*
**Lymphocytes, ×10** ^**9**^ **/L**	0.206	0.046*
**Ferittin**	0.241	0.348

**Fig. 2:**
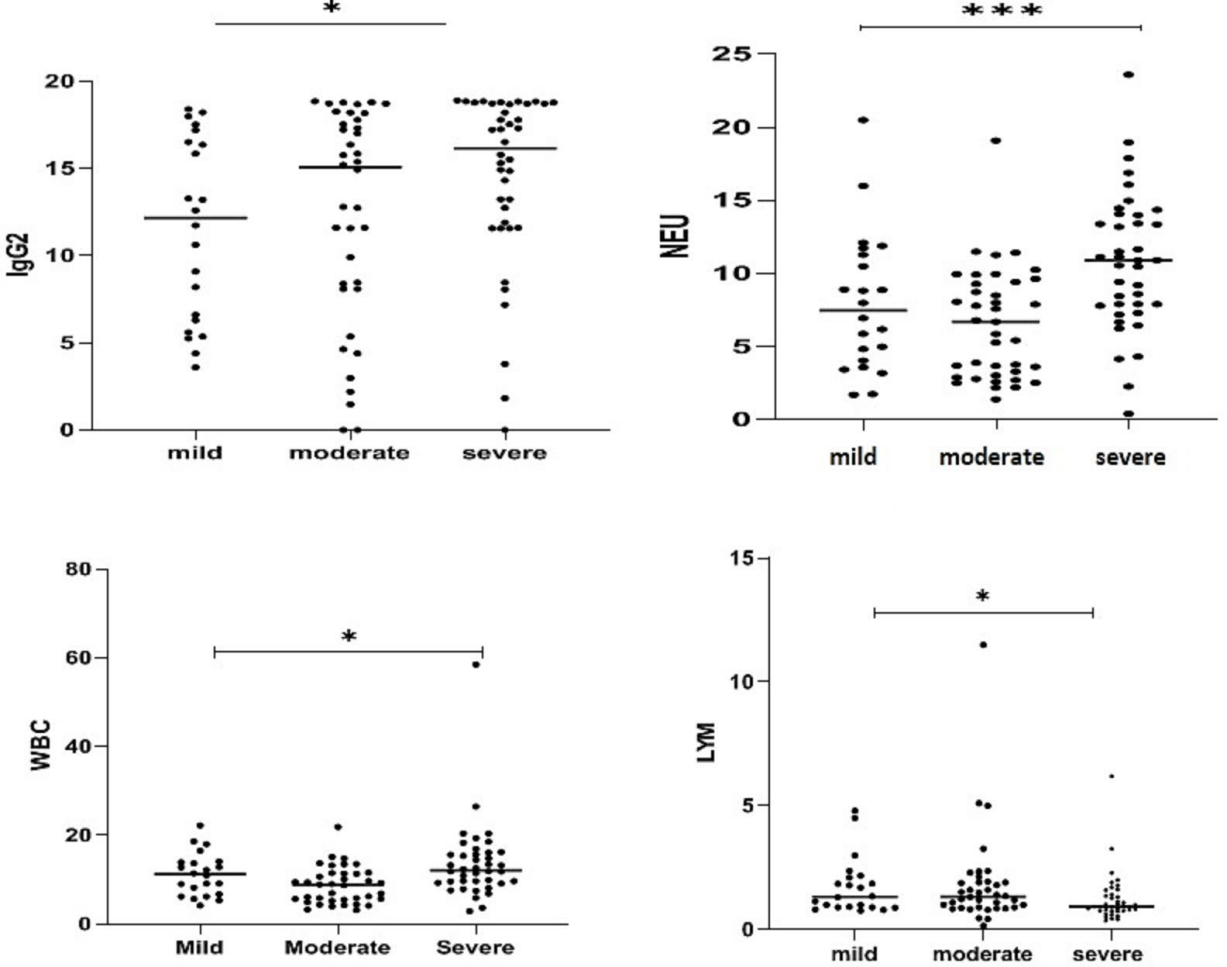
The data showed that the IgG levels in severe group was significantly higher than mild groups. Note: *p <.05 was considered statistically significant.

Interestingly, we found some abnormalities laboratory findings in the sever groups and non-severe groups. Our data showed that the IgG levels in severe group was significantly higher than mild groups. This also included higher WBC, higher neutrophil and lower lymphocyte counts among the groups (*p* < 0.05) (Fig. 2).

## Discussion

The COVID-19 pandemic has focused attention to crucial role of diagnostic techniques in controlling infectious diseases. The standard current diagnostic methods used for SARS-CoV-2 infection based on the stage of disease are nucleic acid-based molecular tests (RT-PCR) and antibody-based tests (Serologic tests). The RT-PCR is used for the early detection of the infection and target SARS-CoV-2 N gene and ORF1ab, while serological test is applied for assessing the disease progression [31, 32]. After SARS infection, IgM antibodies are produced by immune cells during the early stages of infection, followed by IgG generated in the later stages of SARS-CoV-2 infection. The detection of IgM antibody indicates a recent exposure to SARS-CoV-2 and the detection of IgG antibody in the absence of detectable IgM antibody, indicates prior virus exposure [13, 16]. IgG immunoglobulins are monomeric antibodies in the serum and crucial in maintaining long-term immunity or immunological memory after infection [33]. In general, IgM is detectable after 3–6 days, and IgG is detectable after 8 days [34], while viral RNA may be undetectable even after two weeks due to its rapidly decreased level [12]. Therefore, the IgM and IgG antibodies become the main and most accurate procedure to detect an active SARS-CoV-2 infection or even resolved after two months [12, 16]. In this study, we assessed the clinical features and the changed levels of IgG and IgM in 100 COVID-19 patients categorized into mild, moderate and sever groups. All patients showed high specific IgG level which suggested they infected with SARS-CoV-2. According to our results, there was a significant relationship between some clinical symptoms including obesity, fever, and shortness of breath, muscle soreness, odor disorder and taste disorder with the disease severity in COVID-19 patients. However, we found no significant association between age, sex, blood group, some underlying disease including diabetic, hypertension, cardiovascular disease and asthma disease with disease severity. In contrast with our results, a previous study performed by Sotgiu et al. reported a significant correlation between age and sex of COVID-19 patients with severity of the illness. They showed that IgM antibody was dramatically increased in patients in the age groups 20–29 years and 60–69 years compared with those aged from 30 to 59 years. Also, they found a statistically significant higher IgM in males than in females (24.3% VS. 9.1%), showing males were at highest risk of infection and severe disease [33]. Furthermore, our data showed a significantly association between IgG level and severity of disease. The IgG level was found to be significantly higher in severe group than mild group, two months after admission. Thus, the sever patients with higher level of IgG had better and longer-term immunity within weeks or months after infection compare to mild and mediated groups. Our results were comparable with several previous findings of SARS-CoV infections. In compliance with our finding, a previous MERS-CoV study showed that the levels of IgM and IgG antibodies were higher in sever patients compared to patients with mild infection [35, 36]. More studies by Qu and zho et al. reported the delayed IgG and IgM antibody responses as well as higher level of IgG in the critical group compared to non-critical groups [17, 37]. Xie et al. found a higher IgG level in severe than non-sever groups. They also demonstrated a weak correlation between IgM and NEU% percent [37]. In another study done by Park et al. on MERS-CoV, they found that level of IgM antibody response was correlated with reduced disease severity in infected patients [38]. In contrast with our results, Hou revealed that SARS-CoV-2-specific IgM levels were higher and IgG levels were lower in patients in the critical group. While, in the mild group patients compared with the other groups, IgG was maintained at a high level and IgM levels gradually decreased probably due to a compromised immune response in these patients [26]. Moreover, in our study, some laboratory abnormalities including increased levels of ferritin, D-dimer level, LHD, AST, ALT and ALP, decreased fibrinogen levels, lower LYM counts, higher WBC and NUT count were observed in most patients after admission in hospital (step1) compare to two months after admission for COVID-19 (step2). In companion between sever and non-sever groups (mild/moderate), we found a significant higher level of IgG in sever than mild group which was significantly associated with the higher WBC, NUT counts and lower LYM counts (*p* < 0.05). Thereby, the increased titers of anti-virus antibody IgG had a positive association with increased severity of the disease (*p* < 0.05), also longer immunity time after infection in sever patients compared to mild/moderate patients. More interesting that the decreased level of platelets after admission in hospital (step1) compare to two months after admission for COVID-19 (step2) was significantly correlated with the disease severity. In this way, the decreased value of platelets in baseline (step 1) was statistically higher and more significant in moderate to severe groups (*p* < 0.056, *p* < 0.023, respectively) than those in mild group (*p* < 0.974). This finding is likely to be related to the elevated serum D-dimer and changed fibrinogen levels after admission, also blood coagulation and the incidence of strokes in patients with COVID-19 months after recovery.

## Conclusion

Taken together, we concluded that serological markers particularly level of IgG, as the most important anti-COVID-19 antibody response in infected patients can be used for the diagnosis of active or cured COVID-19. In spite of the high abundance of the N protein which makes it a promising candidate for diagnostic serological assays, the its low specificity due to cross-reactivity with other prevalent CoVs may be a critical limitation to its use. Also, in RT-PCR, the results from the two pairs of primers do not agree with each other and the result needs to be re-tested [39].

## Data Availability

The primary data for this study is available from the authors on direct request.
